# Antidepressive effect of an inward rectifier K^+^ channel blocker peptide, tertiapin-RQ

**DOI:** 10.1371/journal.pone.0233815

**Published:** 2020-11-13

**Authors:** Masayoshi Okada, Ikkou Kozaki, Hiroyuki Honda

**Affiliations:** 1 Department of Medical Life Science, College of Life Science, Kurashiki University of Science and the Arts, Kurashiki, Okayama, Japan; 2 Department of Biomolecular Engineering, Graduate Schoosl of Engineering, Nagoya University, Nagoya, Japan; University of Modena and Reggio Emilia, ITALY

## Abstract

Renal outer medullary K^+^ channel, ROMK (Kir1.1, kcnj1) is expressed in the kidney and brain, but its role in the central nervous system remains unknown. Recent studies suggested an involvement of the ROMK channel in mental diseases. Tertiapin (TPN) is a European honey bee venom peptide and is reported to selectively block the ROMK channel. Here, we have chemically synthesized a series of mutated TPN peptides, including TPN-I8R and -M13Q (TPN-RQ), reported previously, and examined their blocking activity on the ROMK channel. Among 71 peptides tested, TPN-RQ was found to block the ROMK channel most effectively. Whole-cell patch-clamp recordings showed the essential roles of two disulfide bonds and the circular structure for the blockade activity. To examine the central role, we injected TPN-RQ intracerebroventricularly and examined the effects on depression- and anxiety-like behaviors in mice. TPN-RQ showed an antidepressive effect in tail-suspension and forced swim tests. The injection of TPN-RQ also enhanced the anxiety-like behavior in the elevated plus-maze and light/dark box tests and impaired spontaneous motor activities in balance beam and wheel running tests. Administration of TPM-RQ suppressed the anti-c-Fos immunoreactivity in the lateral septum, without affecting immunoreactivity in antidepressant-related nuclei, e.g. the dorsal raphe nucleus and locus coeruleus. TPN-RQ may exert its antidepressive effects through a different mechanism from current drugs.

## Introduction

Major depression is a mood disorder with high prevalence and is mostly treated with antidepressants, such as selective serotonin reuptake inhibitors (SSRI) and serotonin-norepinephrine reuptake inhibitors (SNRI). However, according to the STAR*D study, conducted by the American National Institute of Mental Health, current drugs are ineffective in 30% of patients with major depression [1]. Therefore, new medicines, of which the mechanism of action is different from current ones, are desired. It is noteworthy that neurostimulation therapies, i.e., electric convulsive therapy, deep brain stimulation, and transcranial magnetic stimulation, are effective in some drug-resistant depression [2, 3]. It is assumed that the neuronal activities of some nuclei are dysregulated in patients: these neurostimulation therapies compensate for the dysregulated neuronal activity. If they do so, we expect that the pharmacological modulation of neuronal activity may also compensate for the dysregulated activity and be a new treatment for major depression.

K^+^ channels consist of 76 types that modulate the intrinsic excitability of neurons as well as other kinds of cells [4–7] and are classified into three types according to their number of transmembrane domains and opening features, i.e., tandem pore domain, voltage-gated, and inwardly rectifying potassium channels [8]. The inward rectification is a feature that these channels conduct current more easily in the inward direction. The degree of inward rectification varies within inwardly rectifying potassium channels. For instance, whereas Kir2.1, which is referred as strong inward rectifier, conducts much less outward current, the renal outer medullary K^+^ (ROMK; Kir1.1, kcnj1) channel, which is referred as weak inward rectifier, conducts outward current almost as same as the inward current, showing little voltage-dependency [9]. ROMK is expressed in the apical membrane of the Henle loop and collecting duct of the kidney. The channel plays a pivotal role in the aldosterone-sensitive secretion of K^+^ ions, which is coupled to the reabsorption of Na^+^ ions in the kidney [10, 11]; therefore, it is a putative drug target for hypertension without causing hypokalemia [12].

K^+^ channels are widely expressed in the central nervous system, but their roles in the brain are largely unknown. Recent studies have suggested the involvement of K^+^ channels in mental illness. For instance, mutations in a K^+^ channel resulted in a bipolar disorder [13], and some K^+^ channel modulators successfully showed an antidepressive effect in mice [14, 15]. ROMK channel is also expressed in the brain, but its role in the central nervous system remains unknown. Recent studies suggested a possible role of the ROMK channel in mental illness. For instance, the expression of ROMK mRNA was increased 2.54-fold in patients with major depression [16]. A meta-analysis of a genome-wide study found linkage of the ROMK gene with the personality trait of openness [17].

**Fig 1:**
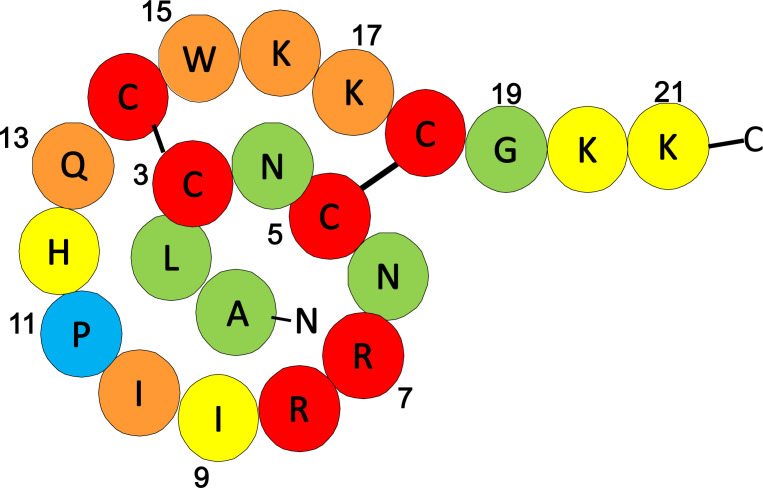
Structure of TPN-RQ. The Amino acid sequence and disulfide bonds of TPN-RQ are illustrated. Each amino acid was classified into five groups and shown in different colors in order of reduction in ROMK blocking activity when each amino acid was changed to Ala (red >80%; orange, 60–80%; yellow, 40–60%; green, 20–40%; blue, <20% at 1 μM) (see text and Table 1).

Various K^+^ channels are blocked by venom peptides [18]. Furthermore, peptide engineering studies have successfully created more specific, potent, and stable peptides than original ones. Tertiapin (TPN) is a peptide purified from European honey bee venom and shown to block ROMK and G-protein activated inwardly rectifying K^+^ (GIRK) 1, 2, and 4 channels (Kir3.1, 3.2, and 3.4, kcnj3, 6, and 5) [18, 19]. TPN consists of 21 amino acids and has two disulfide bonds within the molecule, forming a ring-like structure (Fig 1). In previous studies, because the thirteenth residue of TPN, Met, was easily oxidized, leading to a reduction in the affinity to the channel. a mutation to glutamine (TPN-Q) was introduced to prevent this oxidation [20]. In addition, a computer simulation study showed that the I8R mutation of TPN-Q (TPN-RQ) possibly increased the affinity to Kir2.1 [21], which was also reported to be involved in depression-like behavior [22]. Likewise, peptide engineering studies have successfully created peptides with higher affinity and/or specificity using mutations and shortening of amino acids. For instance, a partial peptide of spadin, PE22-28, showed higher selectivity to a two-pore domain K^+^ channel, TREK-1, rather than the full-length spadin peptide [14].

**Table 1:** ROMK blocking activities of partial and full length TPN peptides.

Peptide #	Group	Sequence	Description	Activity
**1**	Original	ALCNCNRIIIPHQCWKKCGKK	TPN-Q	++++
**2**		ALCNCNRRIIPHQCWKKCGKK	TPN-RQ	++++
**3**		ALCNCNRRIIPHQCWKKCGKK	TPN-LQ	+++
**4**	A	ALCNCNRI	TPN 1–8	-
**5**		LCNCNRII	TPN 2–9	-
**6**		CNCNRIII	TPN 3–10	-
**7**		NCNRIIIP	TPN 4–11	-
**8**		CNRIIIPH	TPN 5–12	-
**9**		NRIIIPHM	TPN 6–13	-
**10**		RIIIPHMC	TPN 7–14	-
**11**		IIIPHMCW	TPN 8–15	-
**12**		IIPHMCWK	TPN 9–16	-
**13**		IPHMCWKK	TPN 10–17	-
**14**		PHMCWKKC	TPN 11–18	-
**15**		HMCWKKCG	TPN 12–19	-
**16**		MCWKKCGK	TPN 13–20	-
**17**		CWKKCGKK	TPN 14–21	-
**18**		RIIIPHQC	TPN-Q 7–14	-
**19**	Partial TPN peptides (8 amino acids)	RRIIPHMC	TPN-R 7–14	-
**20**		RRIIPHQC	TPN-RQ 7–14	-
**21**		RIIIPHQK	TPN-Q 7–13 + K	-
**22**		RRIIPHQK	TPN-RQ 7–13 + K	-
**23**		ARIIPHQK	TPN-RQ 7-13(R7A) + K	-
**24**		RAIIPHQK	TPN-RQ 7-13(R8A) + K	-
**25**		RRAIPHQK	TPN-RQ 7-13(I9A) + K	-
**26**		RRIAPHQK	TPN-RQ 7-13(I10A) + K	-
**27**		RRIIAHQK	TPN-RQ 7-13(P11A) + K	-
**28**		RRIIPAQK	TPN-RQ 7-13(H12A) + K	-
**29**		RRIIPHAK	TPN-RQ 7-13(Q13A) + K	-
**30**		RRIIPHQA	TPN-RQ 7-13(H12A) + A	-
**31**		RIIIPHMC	TPN 7–13 + C	-
**32**		RIIIPHQC	TPN-Q 7–13 + C	-
**33**		RRIIPHMC	TPN-R 7–13 + C	-
**34**		RRIIPHQC	TPN-RQ 7–13 + C	-
**35**	B	CNRRIIPHQC	TPN-RQ 5–14	-
**36**		CNRRIIPKQC	TPN-RQ 5-14(H12K)	-
**37**		CNRRIIPHQCWKK	TPN-RQ 5–17	-
**38**		CNRRIIPKQCWKK	TPN-RQ 5-17(H12K)	-
**39**	Partial TPN-RQ	NCNRRIIPHQCWKK	TPN-RQ 4–17	-
**40**	(10–15 amino acids)	NCNRRIIPKQCWKK	TPN-RQ 4-17(H12K)	-
**41**	with single SS bond	CNSNRRIIPHQCWK	TPN-RQ 3-16(C5S)	-
**42**		CNSNRRIIPKQCWK	TPN-RQ 3-16(C5S/H12K)	-
**43**		QCNSNRRIIPHQCWK	Q + TPN-RQ 3-16(C5S)	-
**44**		QCNSNRRIIPKQCWK	Q + TPN-RQ 3-16(C5S/H12K)	-
**45**	C	ALSNCNRRIIPHQCWKKCGKK	TPN-RQ(C3S)	-
**46**		ALCNSNRRIIPHQCWKKCGKK	TPN-RQ(C5S)	-
**47**		ALCNCNRRIIPHQSWKKCGKK	TPN-RQ(C14S)	-
**48**	Full-length TPN with Cys-to-Ser mutation	ALCNCNRRIIPHQCWKKSGKK	TPN-RQ(C18S)	-
**49**		ALSNCNRIIIPHQSWKKCGKK	TPN-Q(C3S/C14S)	-
**50**		ALSNCNRRIIPHQSWKKCGKK	TPN-RQ(C3S/C14S)	-
**51**	D	ALCNCNRIIPHQCWKKCGKK	TPN-RQ(Δ8R)	++
**52**		ALCNCNRIPHQCWKKCGKK	TPN-RQ(Δ8R/ΔI9)	-
**53**	TPN-RQ smaller or larger ring	ALCNCNRRPHQCWKKCGKK	TPN-RQ(ΔI9/ΔI10)	-
**54**		ALCNCNRRIIIPHQCWKKCGK	TPN-RQ(Ins9I)	-
**55**	E	AACNCNRRIIPHQCWKKCGKK	TPN-RQ(L2A)	+++
**56**		ALCACNRRIIPHQCWKKCGKK	TPN-RQ(N4A)	+++
**57**		ALCNCARRIIPHQCWKKCGKK	TPN-RQ(N6A)	+++
**58**		ALCNCNAAIIPHQCWKKCGKK	TPN-RQ(R7A/R8A)	-
**59**		ALCNCNRAIIPHQCWKKCGKK	TPN-RQ(R8A)	+++
**60**		ALCNCNRRAIPHQCWKKCGKK	TPN-RQ(I9A)	++
**61**	Full-length TPN-RQ peptides with mutations to Ala	ALCNCNRRIAPHQCWKKCGKK	TPN-RQ(I10A)	+
**62**		ALCNCNRRIIAHQCWKKCGKK	TPN-RQ(P11A)	++++
**63**		ALCNCNRRIIPAQCWKKCGKK	TPN-RQ(H12A)	++
**64**	with two SS bonds	ALCNCNRRIIPHACWKKCGKK	TPN-RQ(Q13A)	+
**65**		ALCNCNRRIIPHQCAKKCGKK	TPN-RQ(W15A)	+
**66**		ALCNCNRRIIPHQCWAACGKK	TPN-RQ(K16A/K17A)	+
**67**		ALCNCNRRIIPHQCWKKCAKK	TPN-RQ(G19A)	+++
**68**		ALCNCNRRIIPHQCWKKCGAK	TPN-RQ(K20A)	++
**69**		ALCNCNRRIIPHQCWKKCGKA	TPN-RQ(K21A)	++
**70**	F	CNCNRRIIPHQCWKKCGKK	TPN-RQ(Δ1A/Δ2L)	+++
**71**	N-, C-terminal deletion	ALCNCNRRIIPHQCWKKCG	TPN-RQ(Δ20K/Δ21K)	+

ROMK channel blocking activities of various TPN peptides were examined at varied concentrations and are fitted to the Hill equation (four parameters). The percentage of blockade at 1 μM was estimated according to the fitted curve (-, <20%; +, 20–40%; ++, 40–60%; +++, 60–80%; ++++, >80%). Description (Δ, deletion; Ins, insertion; +, Attached to N- or C-termini).

In this report, to reveal the central role of the ROMK channel, we examined the effect of TPN-RQ in mouse behavioral tests and found antidepressive and anxiogenic effects. TPN-RQ suppressed the neuronal activity, i.e. c-Fos immunoreactivity, in the lateral septum, without affecting those of the dorsal raphe nucleus or locus coeruleus, suggesting that the mechanism of action is different from the enhancement of monoaminergic transmission. We synthesized systematically mutated TPN-RQ and examined the changes in the ROMK blocking ability and found the importance of the disulfide bonds for the blockade.

## Materials and methods

### Peptide synthesis

Peptides were synthesized by a cellulose membrane-based spot-synthesis method a peptide array according to our previous report [23]. A cellulose membrane (grade 542; Whatman, Maidstone, UK) was activated using β-alanine as the N-terminal basal spacer. An Fmoc-Photo-Linker (sc-294977A, Santa Cruz, Dallas, TX) served as a photo-cleavable linker for Fmoc peptide synthesis. The linker conjugated candidate peptides with cellulose. First, the Fmoc-Photo-Linker was activated by mixing with the condensing agents and followed by spotting onto the membrane using a peptide auto-spotter (ASP222; Intavis, Cologne, Germany). Three consecutive steps, including deprotection of the Fmoc group using 20% piperidine, spotting of the Fmoc-activated amino acid on the membrane, and blocking of the remaining amino groups by acetic anhydride were repeated for the elongation of the peptides. The membrane was washed thoroughly with N,N’-Dimethylformamide and followed by methanol. After elongation, the side chain-protecting groups were removed for 2.5 h by a mixture of trifluoroacetic acid (A00025; Watanabe Chemical Industry, Hiroshima, Japan), m-cresol (034–04646; Wako Pure Chemicals, Osaka, Japan), 1,2-ethanedithiol (A00057; Watanabe), and thioanisole (T0191; Tokyo Chemical Industry, Tokyo, Japan) at a ratio of 40:1:3:6. Finally, the membrane was washed thoroughly with diethyl ether and methanol in a consecutive manner. For the oxidation of the thiol group of Cys residues, the peptide arrays were incubated twice with 20% DMSO in phosphate-buffered saline (PBS) (pH = 7.2–7.4) for 24 h at room temperature [24], and washed with PBS. Fmoc-Photo-Linker was cleaved by U.V. (365 nm) irradiation for 3 h using a transilluminator (DT-20LCP; Atto, Tokyo, Japan). Finally, the peptide was eluted from the membrane with 150 μL of PBS by vortexing at room temperature for 30 min. Then, the peptide concentrations were determined using a BCA protein assay kit.

#### Mass spectrometry of peptides

The formation of disulfide bonds was verified with matrix-assisted laser-desorption ionization time-of-flight tandem mass spectrometry (Bruker Daltonik GmbH, Bremen, Germany). The synthesized peptides were dissolved in matrix solution (saturated α-cyano-4-hydroxycinnamic acid in 0.1% TFA/33% CH3CN). Mass spectra across the mass range of 300–4000 m/z were recorded in linear mode using a mass spectrometer in positive ion mode, and the date were analyzed using flexAnalysis software (version 3.0; Bruker Daltonik GmbH). Protein disulfide isomerase (PDI) was purchased from Takara-Bio (Shiga, Japan) and TPN-RQ peptides were incubated at 25°C for 30 min in the presence of PDI and glutathione (oxidized and reduced) according to the manufacturer’s instruction.

#### High-performance liquid chromatography analysis of peptides

For the examination of the chemical stability, the peptide was analyzed with high-performance liquid chromatography (HPLC, JASCO, Tokyo, Japan) using a cation-exchange column (HiTrap-SP, Cytiva). TPN-RQ 33.0 μM, which was mixed with an equal volume of PBS or mouse serum, was analyzed with HPLC immediately after mixing or after incubation at 37°C for 30 min. Peptides were eluted with a gradient of NaCl from 200 to 2,000 mM in 10 mM Tris-HCl (pH 7.4), monitoring A_230 nm_ with a UV detector (JASCO).

### Patch-clamp recordings

The cDNAs for rat ROMK and mouse GIRK1, 2, and 4 were generously donated by Drs Mikio Hayashi (Kansai Medical University) and David Weaver (Vanderbilt University). The ROMK channels were stably expressed by a cell line, 208–4, which was transduced with lentiviral vectors coexpressing the channel and green fluorescent protein (GFP) as described previously [25]. The GIRK genes, cloned in a pCMV6 plasmid (Origene Technologies Inc, Rockville, MD), were transiently expressed in 293T cells with calcium phosphate transfection. GIRK channel currents were recorded 24 h after transfection.

Cells grown on small cover glasses (3 × 18 mm) were placed in a recording chamber. Whole-cell currents were recorded in Tyrode solution using an Axopatch 200B amplifier (Axon Instruments, Foster City, CA) at 25°C. Tyrode solution contained (in mM): NaCl 140, KCl 5.4, NaH_2_PO_4_ 0.33, CaCl_2_ 2, MgCl_2_ 1, HEPES 5, and glucose 5.5 (pH 7.4 adjusted with NaOH). High K^+^ Tyrode solution (KCL 50 mM, NaCl 95 mM, and others were the same as standard Tyrode solution) was used for the recording of GIRK channels. Patch pipettes pulled from borosilicate glass (Narishige, Tokyo, Japan) were filled with an internal solution containing (in mM): K-aspartate 66, KCl 71.5, KH_2_PO_4_ 1, EGTA 5, HEPES 5, and MgATP 3 (pH 7.4 adjusted with KOH). Recordings were digitized at 10 kHz, and low-pass filtered at 2 kHz. ROMK, Kir2.1, and GIRK currents were evoked with step pulses of 400 msec from -150 mV to 10 mV in 10 mV increments from a holding potential of -70 mV. Whole-cell currents were recorded 60 sec after the peptide application and those from 100 to 300 msec of the step pulses were averaged. Liquid junction potential or capacitive transient was not corrected or compensated. Whole-cell conductance was calculated as the slope of the current-voltage (I-V) relationship from -140 mV to -100 mV and normalized to the initial value (0 μM TPN), in which BaCl_2_-resistant current was subtracted and indicted as a percentage.

### Mouse behavioral tests

We performed mouse behavioral experiments in accordance with our previous study [15]. Male ICR mice (n = 199 in total) aged 5 weeks old were purchased from Shimizu Laboratory Supplies (Kyoto, Japan) and maintained in an animal facility for 7–12 days. They had free access to food and water and were kept in a 12-h light/dark cycle (08:00–20:00). Experiments were carried out between 11:00 and 17:00 in a quiet and air-conditioned experimental room, to which mice were habituated for 30–60 min before each experiment. Animal experiments were performed in accordance with the guidelines of the Physiological Society of Japan and were approved by the Committee for Animal Experiments of Kurashiki University of Science and the Arts. Peptides were dissolved in phosphate-buffered saline (PBS). Peptides were injected intracerebroventricularly (i.c.v.) 30 min before behavioral tests (typically 5 μL/mouse), according to a previous study [26]. The mouse was placed into an anesthetizing jar, to which isoflurane (0.2 mL) was added immediately before. After confirming that the mouse was deeply anesthetized we injected the peptide into the right cerebral ventricle over 30 seconds and the injection needle was kept for 1 min after the injection (i.c.v. coordinates: 0 mm caudal to bregma, 1.0 mm lateral to the midline, and 3.5 mm below the skull). The mouse was placed in a cage set on a heating pad (approximately 33°C) for a few minutes until it spontaneously started to move, and then it was put back into its home cage. Each animal was used only once, except for the wheel running test, which was carried out immediately after the elevated plus-maze.

#### Tail suspension test

The tail suspension test (TST) was performed in accordance with the method reported previously [15]. The mouse was hung on a hook using adhesive tape placed 2 cm from the extremity of its tail 50 cm above a table. The amount of time spent immobile was recorded for 5 min.

#### Forced swim test

The forced swim test (FST) was performed in accordance with the method reported previously [15]. Briefly, a Mouse was placed into a 2 L glass beaker containing 12 cm of water (25–27°C). The mouse was allowed to swim for 6 min and its activity was measured. The duration of immobility was defined as the absence of activity, such as escape-oriented behaviors. The last 4 min of data were used for analysis. A mock experiment was performed 1 day before the real test.

#### Novelty suppressed feeding test

Mice were caged singly, and food was removed from the cage for 24 h before the novelty suppressed feeding (NSF) test, although water was available *ad libitum*. A piece of a familiar food pellet was placed on a white circular filter paper (12.5 cm in diameter), which was placed at the center of the open field. A mouse was placed into a corner of the open field 30 min after i.c.v. The latency to beginning to feed and food consumption were measured for 5 min.

#### Balance beam test

Motor coordination was assessed by measuring the ability to traverse a narrow beam to reach a familiar cage. We used wooden beams 90 cm in length and of several widths (22, 12, 10, and 6 mm). The beams were placed horizontally, 30 cm above the bench surface with one end mounted on a familiar cage. The experiment was preceded by 3 days of training: the mouse was trained twice with a 22 mm beam on the first training day and with 12 and 10 mm beams on the second and third day, respectively. On the trial day, mice were subjected to two consecutive trials with a 10 mm beam, followed by two trials with a 6 mm beam. The time required to cross the central 60 cm of the beam was measured.

#### Wheel running test

Spontaneous motor activity was measured with a multifunctional activity wheel (MK-770, Muromachi Kikai, Tokyo, Japan). Immediately after the EPM test, the mouse was placed into the wheel and allowed to run freely for 4 min. The number of rotations was counted automatically.

#### Open field test

The open-field test (OFT) was performed in accordance with a previous report [15]. The open-field consisted of a square (90 × 90 cm), in which the floor was divided into 16 equal squares. After a mouse was placed in a corner of the open field facing the wall, the number of the entries into the central squares and whole line crossings were counted for 5 min. After the removal of the animal, the apparatus was cleaned and wiped.

#### Elevated plus-maze

The elevated plus maze (EPM) apparatus consisted of four arms set in a cross pattern from a neutral central square (6 × 6 cm). Two opposite arms were delimited by vertical walls (closed arms, 30 × 15 × 6 cm), whereas the other two arms had unprotected edges (open arms, 30 × 6 cm). The maze was elevated 40 cm above the floor. At the beginning of each 5 min test session, a mouse was placed in the central neutral area, facing one of the open arms. The total number of entries to open arms and time spent in the open arms was measured. After the removal of the animal, the apparatus was cleaned and wiped.

#### Light/dark box test

The light/dark box test (LDT) consisted of two compartments with a total exterior size of 46 × 27 × 30 cm. One-third of the box was used as a dark compartment, which was covered with a black wall and lid, and the rest of the area was used as a light compartment. These compartments were connected via a small opening (7 × 7 cm), enabling transition between the two compartments. We placed a mouse at the center of the light area and measured the time spent in the light area and the number of transitions between the light and dark compartments for 5 minutes.

#### Chronic unpredictable mild stress

Chronic unpredictable mild stress (CUMS) procedure was conducted according to previous studies with some modifications [27, 28]. Mice underwent one of the following stressors in random order for 18 days: constriction (30 min, in 50 mL conical tube), forced swimming (6 min), cage oscillation (30 min, 100 rpm horizontal and reciprocal movement), cage tilting (1 hr, 45°), Barne’s maze (up to 5 min or run into the escape box two times), tail suspension (5 min), and elevated plus maze (5 min). The number of each stressor was 4, 4, 3, 3, 2, 1, and 1, respectively. These stressors were given at the varied times of day (from 11 AM to 4 PM) making them unpredictable. On the 19th day, depression-like behavior was tested with TST 30 min after i.c.v. of TPN-RQ or PBS.

### Anti-c-Fos immunostaining

Thirty min after being subjected to the TST, the mouse was anesthetized as above. After confirming that the mouse was deeply anesthetized, the brain was fixed by transcardiac perfusion with 4% paraformaldehyde in PBS. The brains were sliced coronally (100 μm in thickness) using a microslicer (PRO7, Dosaka, Kyoto, Japan). The free-floating slices were reacted with an anti-c-Fos antibody (1: 200, goat, Santa Cruz, Dallas, TX) dissolved in PBS containing 0.3% BSA and 0.3% Triton-X-100, at 4°C for 48 h. The immunoreaction was visualized using a secondary antibody (rabbit anti-goat IgG, Vectastain Elite ABC-HRP Kit, Vector Labs, Burlingame, CA) and 3,3'-Diaminobenzidine (DAB; Tokyo Chemical Industry, Tokyo, Japan) at room temperature in accordance with the manufacturer's instructions. For statistical analysis, the number of c-Fos-immunoreactive cells was quantified using Image-J software. Briefly, fields (0.16 mm^2^) of c-Fos immunoreactivity were acquired using a microscope using a CCD camera. DAB-positive nuclei, of which the diameters were longer than 7 μm, were considered as anti-c-Fos-immunoreactive neurons. The immunoreactive cell numbers were normalized to cells/mm^2^.

### Statistical analysis

Data are given as the mean ± SEM. The normality of data distribution was confirmed with the Shapiro-Wilk test. Statistical significance between two groups was determined using Student's t-test. When data are not normally distributed, significance was determined with Kolmogorov-Smirnov test. Those obtained from three or more groups were analyzed statistically by one-way analysis of variance (ANOVA) followed by Bonferroni test. A *p* value of <0.05 was considered significant. The number of asterisks indicates the *p* values: *, *p* < 0.05; **, *p* < 0.01; ***, *p* < 0.005.

## Results

### ROMK blocking activity of TPN-RQ

**Fig 2:**
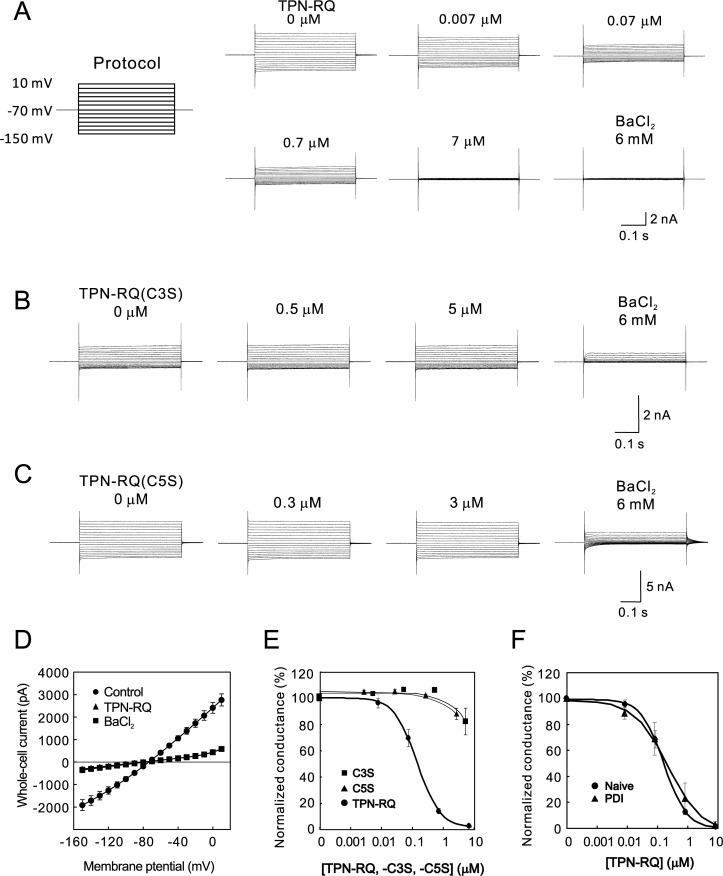
Blockade of ROMK currents by TPN-RQ and the lack of effect of Cys-to-Ser mutants. (A) Whole-cell ROMK currents were evoked by step-pulses, as shown in the protocol. The application of TPN-RQ decreased ROMK currents in a concentration-dependent manner. The remaining current was resistant to 6 mM BaCl_2_, which is a selective blocker of the ROMK channel, indicating that TPN-RQ blocked the channel nearly completely. (B and C) The C3S and C5S mutants of TPN-RQ hardly inhibited the ROMK currents even at the highest concentrations, indicating the essential role of these Cys residues. (D) I-V relationship of whole-cell ROMK channel currents in the absence (Control) or presence of blockers (7 μM TPN-RQ and 6 mM BaCl_2_). The I-V curves of TPN-RQ and BaCl_2_ were completely overlapped. (E) The concentration–response curve of TPN-RQ, (C3S), and (C5S) on ROMK conductance (n = 3). (F) Preceding treatment of TPN-RQ with PDI did not affect the blocking activity of TPN-RQ, suggesting the adequate formation of disulfide bonds (n = 4).

To analyze the central role of the ROMK channel and to reveal the structure and function relationship between TPN and ROMK channel blocking activity, we chemically synthesized TPN and related peptides. Because the methionine residue at position 13 of the original peptide was sensitive to oxidation, this residue was changed to glutamine (TPN-Q). We also synthesized an I8R mutant (TPN-RQ), which was mainly used in this study because of its highest activity as shown in the latter part of this paper. For the patch-clamp recordings of ROMK channel currents, we prepared a 293T cell line, 208–4, which stably coexpressed ROMK and GFP using the lentiviral vector. We recorded whole-cell current from the 208–4 cells and observed the weakly inwardly rectifying without showing obvious voltage-dependency (Fig 2A and 2D). This feature of whole-cell current was the same as the previous report [9]. We then tested the effect of peptides on channel conductance. As expected, the application of TPN-RQ decreased the ROMK current in a dose-dependent manner, with an IC_50_ of 0.17 μM (Fig 2A and 2E). TPN-RQ (7 μM) completely abolished the BaCl_2_-sensitive currents at from -150 to 10 mV, indicating the voltage-independence of blocking activity (Fig 2D). In contrast, two Cys to Ser mutants (TPN-C3S and -C5S), which lacked one of the Cys residues required for the two disulfide bonds, showed little blocking activity (Fig 2B, 2C, and 2E); therefore, they served as negative control hereafter.

After chemical synthesis, we confirmed the formation of the two disulfide bonds in TPN-RQ with MS analysis: the m/z value of the main peak was 2494.2, which corresponded to the estimated value with two disulfide bonds, 2495.1. However, it was also possible that these disulfide bonds were formed between incorrect pairs of Cys residues. To exclude this possibility, the TPN-RQ peptides were treated with protein disulfide isomerase (PDI), which rearranges the disulfide bonds [29]. Subsequently, blocking activities of naïve and PDI-treated TPN-RQ were examined with whole-cell recordings from 208–4 cells (Fig 2F). PDI treatment did not affect the blocking activity (IC_50_ 0.24 μM), suggesting the appropriate formation of disulfide bonds in the synthesized TPN-RQ.

**Fig 3:**
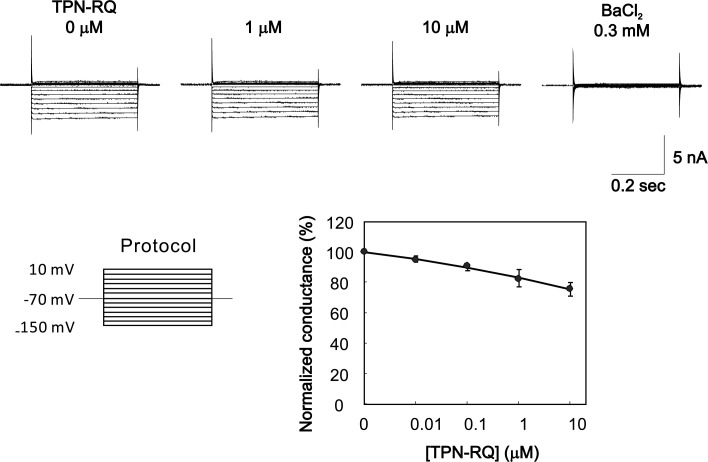
Lack of effect of TPN-RQ on the Kir2.1 channel. Whole-cell currents were recorded from 56–3 cells, and Kir2.1 channel currents were evoked by the step pulses. TPN-RQ hardly inhibited the 0.3 mM BaCl_2_-sensitive Kir2.1 currents.

A previous computer simulation study suggested that RQ mutation of TPN can lead to blockade of Kir2.1 at a nM range, as well as ROMK [21]. To test this possibility, we made whole-cell recordings from a Kir2.1 expressing cell line, 56–3 [30], and examined the effect of TPN-RQ (Fig 3). Unexpectedly, TPN-RQ hardly blocked the Kir2.1 channel current even at 10 μM.

### Antidepressive effect of TPN-RQ

**Fig 4:**
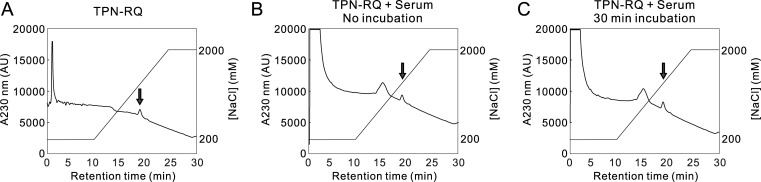
Chemical stability of TPN-RQ. Chemically synthesized TPN-RQ (100 μL, 16.5 μM) was fractionated on a cation exchange column, monitoring A_230 nm_ (A). A peak was found with a retention time of 17.5 min on the declining baseline (arrow). Then, the peptides, which were mixed with mouse serum, were analyzed (B and C). The incubation with serum did not affect the peak height or retention time. The ordinate indicates the A_230 nm_ (arbitrary unit of HPLC UV-detector) and NaCl concentration (mM).

Before the behavioral test, we tested the chemical stability of the peptide: TPN-RQ was incubated with an equal volume of mouse serum at 37°C for 30 min and subsequently analyzed with HPLC (Fig 4). No changes in the peak height or retention time were observed, indicating the chemical stability against proteolytic degradation.

**Fig 5:**
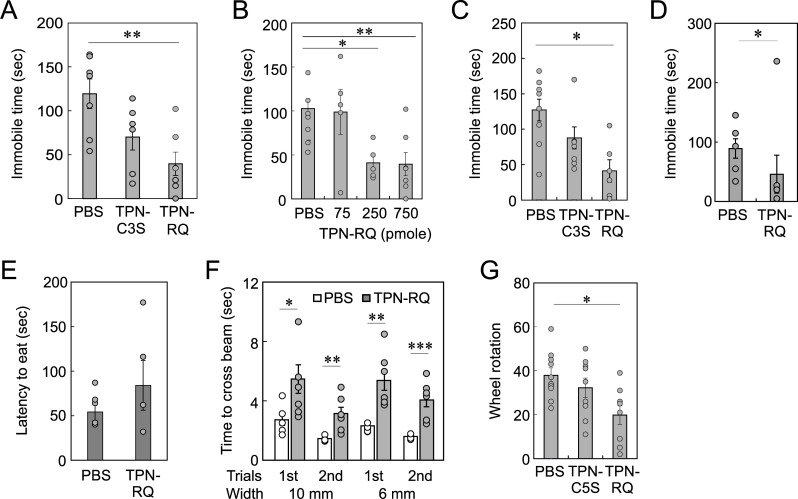
Antidepressive effect of TPN-RQ. (A) TPN-RQ decreased immobile time at the TST, whereas TPN-RQ(C3S) decreased the time insignificantly. (B) Dose-dependency of TPN-RQ on immobile time in the TST. (C) TPN-RQ, but not TPN-RQ(C3S), decreased the immobile time at FST. (D) Significant decrease in immobile time in the TST of the chronically stressed mice. (E) TPN-RQ insignificantly increased the latency for beginning eating in the NSF test. (F) Impairment of motor coordination by TPN-RQ. The administration of TPN-RQ significantly increased the time required to cross the central 60 cm of the beam in consecutive 4 trials (10 mm beam, 2 trials; 6 mm, 2 trials). (G) TPN-RQ decreased spontaneous rotations in the activity wheel in 4 min.

To reveal the role of the ROMK channel in the central nervous system, we next examined the effect of i.c.v. injection of TPN-RQ on mouse behavior. We first analyzed the effect of TPN-RQ on depression-like behavior in the TST, in which typical SSRIs, e.g. fluoxetine and paroxetine, decreased the immobile time [31]. We administered 750 pmole of TPN-RQ into the right lateral ventricle of ICR mice (6 weeks old) and measured immobile time 30 min after the injection for 5 min. If the peptide was equally distributed throughout the mouse brain, the concentration was estimated to be 1.57 μM, which was higher than the IC_50_ for ROMK channel. The administration of TPN-RQ significantly decreased the immobile time (ANOVA, F(2, 17) = 3.59, *p* < 0.01), suggesting an antidepressive effect (Fig 5A). The C3S mutant, which scarcely blocks the channel, decreased the immobile time insignificantly. This antidepressive effect of TPN-RQ was dose-dependent (Fig 5B): the lower dose (250 pmole) had a similar effect, but the lowest dose (75 pmole) did not affect the immobile time (F(3, 26) = 2.98, *p* < 0.005). To confirm this antidepressive effect, we examined the effect on the FST, in which typical SSRIs decrease the immobile time [32]. TPN-RQ again decreased the immobile time (Fig 5C) (F(2, 19) = 8.703, *p* < 0.01), confirming the antidepressive effect. The TPN-C3S again decreased the immobile time insignificantly.

We then tested the antidepressive effect of TPN-RQ on the mice subjected to chronic unpredictable mild stress (CUMS) with TST. There are some variations in CUMS protocols: reportedly, when mice were subjected to two or more stressors per day, they became more depressive [28], whereas they could adapt to stress when they underwent one stress per day [33]. In this experiment, in which mice were subjected to one mild stress per day, mice seemed to adapt to the stress more or less: the immobile time of PBS-treated mice was insignificantly decreased in CUMS mice (compare the PBS columns in Fig 5A and 5D). The administration of TPN-RQ decreased the immobile time, showing again antidepressive effect to the chronically stressed mice (Fig 5D, Kolmogorov-Smirnov test, *p* < 0.05).

We next examined the effect of TPN-RQ on the novelty suppressed feeding (NSF) test, in which the latency to begin eating was shown to be reduced by chronic treatment with antidepressants and an anxiolytic, diazepam [34]. Unexpectedly, the administration of TPN-RQ insignificantly increased the latency (Fig 5E, Student’s t-test, *p* = 0.30, n = 6). Given, TPN-RQ has antidepressive effect, this nonsignificant increase in latency can be explained with the following possibilities: that TPN-RQ has an anxiogenic effect and/or that TPN-RQ impaired motor coordination, and thereby the antidepressive effect of TPN-RQ was canceled. The latter possibility was likely because the movement of TPN-RQ-treated mice was slow compared with PBS-treated mice, although we did not analyze this statistically. To test this possibility, mice were subjected to a balance beam test. As expected, the time required to cross the beam was longer in TPN-RQ-treated mice (Fig 5F). To confirm the motor impairment, we measured spontaneous rotations in an activity wheel (Fig 5G). TPN-RQ decreased spontaneous motor activity (F(2,27) = 3.37, *p* < 0.05), supporting the impairment of locomotor activity. This motor impairment can exclude the possibility that the decreases in the immobile time in the TST and FST, mentioned above, were due to an increase in gross motor activity, supporting the antidepressive effect of TPN-RQ.

### Anxiogenic effects of TPN-RQ

**Fig 6:**
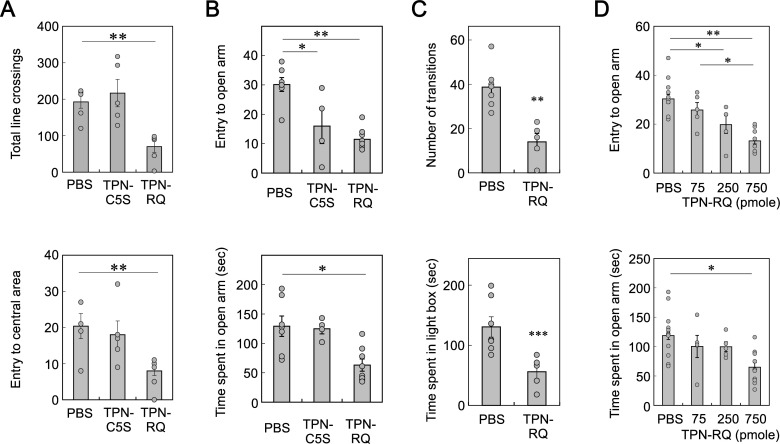
Anxiogenic effect of TPN-RQ. TPN-RQ, but not TPN-RQ(C5S), showed an anxiogenic effect in the OFT (A), EPM (B), and LDT (C). The anxiogenic effect was dose-dependent in the EPM (D).

The increase in latency to begin eating at the NSF test can also be explained by the possible anxiogenic effect of TPN-RQ. To test this, mice were subjected to OFT, EPM, and LDT. In these tests, the administration of typical anxiolytic agents increased exploratory behaviors in aversive area, such as, brightly lit open field (OFT), elevated open arms (EPM), and light compartment (LDT) [35]. In the OFT, TPN-RQ decreased the number of total line crossings (F(2,14) = 3.74, *p* < 0.005) and entries to the central area (F(2,14) = 3.74, *p* < 0.05) (Fig 6A), suggesting that that peptide had an anxiogenic effect too. Mice were then subjected to the EPM (Fig 6B). TPN-RQ administration decreased both the number of entries to the open arms (F(2, 16) = 3.63, *p* < 0.0005) and the time spent in the open arms (F(2, 16) = 3.63, *p* < 0.005), supporting the anxiogenic effect of TPN-RQ. Similarly, consistent results were obtained with the LDT (Fig 6C). TPN-RQ treatment decreased the number of transitions (*p* < 0.001, Student’s t-test, n = 7 and 5) and the time spent in the light area (*p* < 0.01). The anxiogenic effect was dose-dependent in the EPM (Fig 6D) (F(3, 40) = 14.4, *p* < 0.000001; F(3, 40) = 8.12, *p* < 0.0005). The decreases in the numbers of line crossings in the OFT, entry to open arm in the EPM, and transition between areas in the LDT may also be explained by the motor impairment in part. However, the decreases in the time spent in the open arm and light area were more readily explained by the anxiogenic effect.

### Antidepressive and anxiogenic effects of TPN-LQ without affecting the GIRK channel

**Fig 7:**
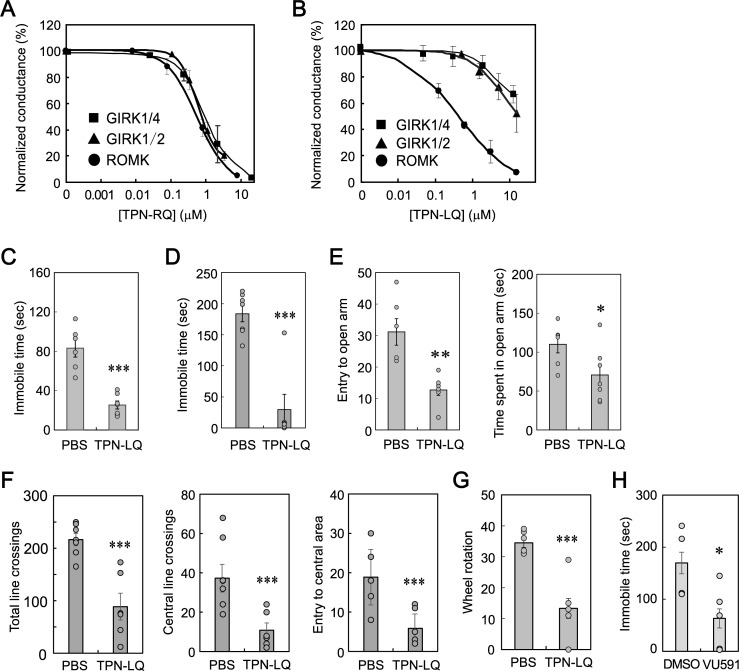
The ROMK-selective blocker TPN-LQ showed similar behavioral effects. (A) TPN-RQ blocked ROMK, GIRK1/2, and GIRK1/4 channels nearly equally. (B) TPN-LQ blocked ROMK channel selectively over GIRK1/2 and GIRK1/4 channels. TPN-LQ decreased immobile time in the TST (C) and the FST (D) at the same dose as TPN-RQ. TPN-LQ showed the anxiogenic effect in EPM (E) and OFT (F). TPN-LQ decreased spontaneous rotations in the activity wheel, indicating the impairment of locomotor activity (G). Another ROMK selective blocker, VU591, also showed antidepressive effect in the TST (H).

Reportedly, TPN-Q blocked GIRK1/2 and 1/4 channels as well as the ROMK channel [19], and GIRK2-deficient mice showed a depression-resistant phenotype [36]. Therefore, it is also possible that TPN-RQ showed its effects through the blockade of the GIRK channel. To exclude this possibility, we synthesized an H12L mutant, TPN-LQ, which was reported to be more selective for the ROMK channel [37]. To confirm the higher ROMK selectivity over the GIRK channel, whole-cell currents were recorded from 293T cells expressing ROMK, mouse GIRK1/2, or GIRK1/4 in the presence of various concentrations of TPN-RQ and -LQ. TPN-RQ inhibited the ROMK, GIRK1/2, and GIRK1/4 conductances with similar concentration-dependency (IC_50_; 0.57, 0.61, and 1.25 μM, respectively) (Fig 7A). In contrast, TPN-LQ selectively blocked the ROMK channel over the GIRK channels (Fig 7B): the difference in the IC_50_ against GIRK1/2 was 20.9 fold (IC_50_; 0.445 and 9.28 μM, respectively). Similarly, TPN-LQ hardly inhibited the GIRK1/4 channel, blocking only 33.1% at 12.7 μM (IC_50_; 2.70 μM).

Then we tested the effect of i.c.v. administration of ROMK-selective TPN-LQ in the TST (Fig 7C). As expected, TPN-LQ decreased the immobile time at the same doses as TPN-RQ (750 pmole) (*p* < 0.00001, Student’s t-test, n = 6 and 7), suggesting the involvement of ROMK, rather than GIRK1/2, in the antidepressive effect. TPN-LQ also decreased the immobile time in the FST (Fig 7D) (*p* < 0.0005, Student’s t-test, n = 6 and 7). Similarly, TPN-LQ decreased the number of entries to open arms and the time spent in open arms in the EPM (Fig 7E) (*p* < 0.005 and 0.05, n = 6 and 7) and the numbers of total line crossings, the central line crossings, and the entry to the central area in the OFT (Fig 7F) (*p* < 0.001, 0.001, and 0.005, n = 6 and 7), suggesting that the anxiogenic effect was also mediated by blockade of the ROMK channel. Wheel rotation was also decreased (Fig 7G) (*p* < 0.0005, n = 6 and 7). To confirm the involvement of ROMK channel in the depression-like behavior, we injected VU591, which was reported to block ROMK channel selectively [38], and examined the effect in TST. As expected, the i.c.v. administration of VU591 (5 μL of 20 mM in dissolved in dimethyl sulfoxide) significantly decreased the immobile time (Fig 7H) (*p* < 0.05, n = 4 and 5).

### A nucleus is involved in the antidepressive effect of TPN-RQ

**Fig 8:**
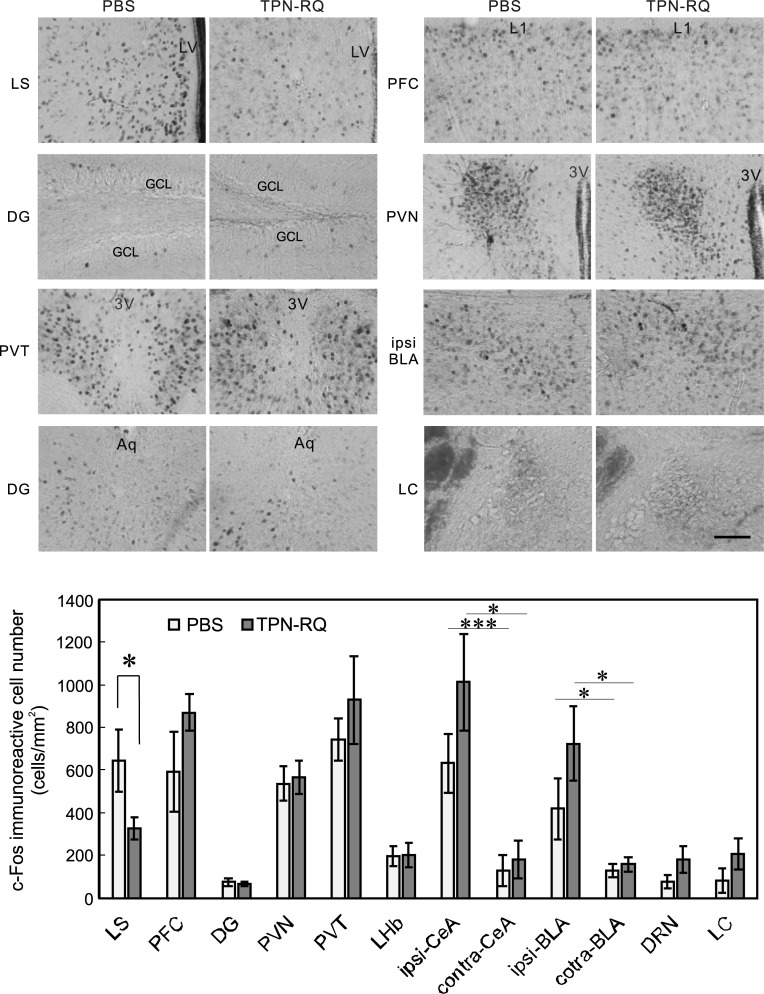
Anti-c-Fos immunoreactive cell numbers in depression-, anxiety-, and stress-related nuclei. A significant difference in the number of anti-c-Fos immunoreactive neurons was observed only in the lateral septum. (LS, lateral septum; PFC, prefrontal cortex (cingulate cortex); DG, dentate gyrus; PVN, hypothalamic paraventricular nucleus; PVT, thalamic paraventricular nucleus; LHb, lateral habenular nucleus; [ipsi, injection side; contra, opposite side] CeA, central amygdala; BLA, basolateral amygdala; DRN, dorsal raphe nucleus; LC, locus coeruleus: LV, lateral ventricle; L1, layer 1; GCL, granule cell layer; 3V, third ventricle; Aq, central aqueduct) (bar = 100 μm, n = 7 and 6).

Given that TPN-RQ changed the neuronal activity of a certain nucleus and thereby changed the behavior of mice, the above findings raised a question about which nucleus is involved in these behavioral effects. If TPN-RQ exhibits antidepressive effects by enhancing the transmission of serotonin and norepinephrine, neuronal activities of the dorsal raphe nucleus and locus coeruleus should be increased [39]. If not, that of another nucleus should be changed. In our previous report [15], we found a drug-responsive change in anti-c-Fos immunoreactivity, which is a marker of neuronal activity [40], in mildly stressed (i.e. being subjected to TST) mice; therefore, we fixed TPN-RQ- or PBS-treated mouse brains at 30 min after TST. Then, we examined the effect on anti-c-Fos immunoreactivity in depression-, anxiety-, and stress-related nuclei. Preceding administration of TPN-RQ tended to increase the numbers of anti-c-Fos immunoreactive neurons in most nuclei, i.e., prefrontal cortex, thalamic paraventricular nucleus, and central and basolateral nuclei of the amygdala, compared with PBS-treated mice (Fig 8). Of note, the changes in anti-c-Fos immunoreactivity were nonsignificant in the dorsal raphe nucleus and the locus coeruleus, which provide serotonergic and noradrenergic innervations (*p* = 0.25 and 0.26, Student’s t-test, n = 7 and 6). Instead, we found a significant change only in the lateral septum: The number of anti-c-Fos immunoreactive neurons was significantly decreased in TPN-RQ-treated mice (*p* < 0.05), suggesting an involvement of this nucleus. We injected the peptide only into the right lateral ventricle, a significant difference was observed in amygdalar nuclei between the ipsilateral side and the contralateral side. Since these differences in the amygdala were also observed in the PBS-treated mice, this side-dependent change was not attributable to TPN-RQ. No significant differences were observed between ipsi- and contralateral sides in other nuclei, including the lateral septum.

### Essential amino acid structures of TPN for ROMK channel blockade

**Fig 9:**
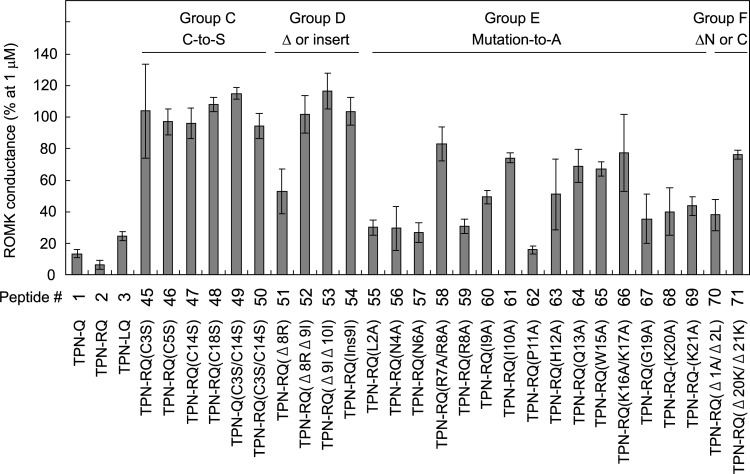
ROMK channel blocking activities of TPN mutants. Ordinate indicates the normalized whole-cell conductances of ROMK channels treated with the indicated peptide at a concentration of 1 μM, which were estimated according to the fitted curve (n = 3–6). When the mutation resulted in the loss of the blocking activity, i.e. the remaining conductance was high, the mutated amino acid was colored red in Fig 1.

To reveal the essential amino acids and structure–function relationship of TPN for ROMK channel blockade, we synthesized a series of mutated TPN peptides and examined the blocking ability of the ROMK channel current. Because a previous study successfully engineered a highly specific blocker of the TREK-1 channel with shortened spadin peptide (7 amino acids long) [14], we first synthesized a series of partial peptides consisted of 8 amino acids without disulfide bonds. Unexpectedly, none of the short peptides had blocking activity (Table 1, Group A, Peptide # 4–20). Because the importance of R7, H12, Q13, and Lys residues at the C-terminus have been reported [18, 41], we attached a Lys residue to residues 7–13 of TPN-Q and -RQ (# 21 and 22), but neither of them again had blocking activity. There might be a hindering amino acid within the partial TPN peptide, mutations to Ala were introduced for each amino acid (# 23–30), but Ala-mutations again resulted in a lack of blocking activity. Similarly, the attachment of a Cys residue to the C-terminus failed to block the channel (# 31–34). We next synthesized partial peptides with a single disulfide bond and a length of 10–15 amino acids and tested the channel blocking activity. These peptides also showed no activity (Table 1, Group B), suggesting the importance of the full-length peptide and/or two disulfide bonds. To test the requirement for the two disulfide bonds, we synthesized the full-length peptide with a single disulfide bond, in which one or two Cys residues were mutated to Ser (Table 1 and Fig 9, Group C). These single disulfide bond peptides had no ROMK blocking activity: for instance, TPN-C3S and -C5S, which were used as the negative control, scarcely blocked the channel. These results indicated the essential role of two disulfide bonds, which make the ring-like structure inflexible. Fig 9 summarizes the percentage of residual conductance treated with each peptide at a concentration of 1 μM.

These results indicated the essential role of the inflexible ring-like structure, we next examined the effect of the size of the ring. We synthesized TPN peptides with a smaller ring by deletion of amino acids and a larger ring by insertion (Group D). The deletion of arginine residue 8 resulted in weak blocking activity, and that of two amino acids abolished the activity (# 51–53). Conversely, the insertion of an extra isoleucine as residue 9 also abolished the activity (# 54). These results suggested that the size of the ring is critical for the blockade.

Finally, to analyze the essential amino acids, we mutated each amino acid to alanine one by one (Group E). Generally, each mutation reduced the blocking activity more or less, except for TPN-RQ(P11A) (# 62) indicating the nonessential role of this residue. Mutations of amino acids that were simulated to be localized at the surface [18], resulted in a larger decrease in the activity. The mutations of basic amino acids (R7A, K16A, K17A, K20A, and K21A) also strongly reduced activity. Similarly, the deletion of C-terminus basic amino acids greatly reduced the blocking activity (Group F, # 71). In contrast, mutations of the neutral N-terminal amino acid residues subtly decreased the activity (# 55–57), and the deletion of these N-terminus amino acid residues modestly reduced the activity (# 70). Among them, TPN-RQ showed the highest blocking activity and followed by TPN-Q. Essential and less important amino acids are shown in different colors (Fig 1). These results indicated that TPN-RQ was highly specific to the ROMK channel.

## Discussion

The ROMK channel is widely expressed in the brain, but its role in mood disorders had remained unknown. Here, we show the antidepressive effects of a ROMK blocker, TPN-RQ, suggesting the involvement of this channel in depression. The antidepressive effect of TPN-RQ was indicated by decreases in immobile times in the TST and FST. In contrast, the negative control, Cys-to-Ser mutants, hardly showed an antidepressive effect, excluding the possibility of a nonspecific effect of i.c.v. administration of peptides. In addition, TPN-RQ was suggested to have an anxiogenic effect and impair locomotor activity in several behavioral tests. The decreases in the number of total line crossings in the OFT, entry to open arm in the EPM, and transitions in the LDT can be explained with both anxiogenic effects and motor impairment. We confirmed that TPN-RQ had these two effects with separate behavioral tests. The impairment of locomotor activity was shown by the increase in latency in the balance beam test. On the other hand, the anxiogenic effect of TPN-RQ was suggested by the decreases in the entry to the central area in the OFT, and time spent in the open arm in the EPM and light area of the LDT, which are all assumed to be hardly affected by motor impairment. The possibility of the involvement of the GIRK2 channel was excluded by the result that TPN-LQ, which is 20.9-fold selective to ROMK over GIRK1/2, showed these effects similarly to TPN-RQ. Deficiency of the ROMK gene leads to type 2 Bartter syndrome, of which the main symptoms are transient hyperkalemia, polyuria, and metabolic acidosis after birth [42]. Moreover, mental abnormalities are also reported in patients with Bartter syndrome, i.e. psychotic and mania-like symptoms [43, 44]. It remained unknown whether these psychotic symptoms are attributable to abnormalities in the serum ion composition or to the functional loss of the ROMK channel in the brain. Our data showed that the blockade of central ROMK channels showed an antidepressive effect in mouse behavioral tests. The increased expression of ROMK mRNA [16] may result in the opposite effect in patients with depression.

Anti-c-Fos immunostaining suggested an involvement of the lateral septum in the antidepressive effect of TPN-RQ, which significantly decreased c-Fos-positive neurons in the nucleus. In contrast, the numbers of anti-c-Fos immunoreactive neurons were increased only insignificantly in the dorsal raphe nucleus and the locus coeruleus. Therefore, the transmission of serotonin and norepinephrine was not thought to be enhanced. These findings suggested a difference in the mechanisms of action from current SSRIs and SNRIs and the presence of a novel mechanism of antidepressive action. Together with previous studies, in which K^+^ channel modulators successfully showed antidepressive effects [14, 15, 45], our data suggested that the modulation of neuronal activity through K^+^ channels is a promising method as antidepression therapy. To that goal, it will be needed to elucidate the nucleus that plays the causative role in the antidepressive effect. Notably, our previous study has shown that a TREK-1 channel activator, ostruthin, which had antidepressive and anxiolytic effects, also decreased anti-c-Fos immunoreactivity in the lateral septum [15], suggesting that these two compounds exhibit antidepressive effects through the suppression of the nucleus. The lateral septum was reported to be involved in freezing behavior and relay the information of stress to other nuclei [40]. Mental stress can lead to depression; therefore, it is expected these compounds may be effective in humans, especially for drug-resistant cases. Moreover, if the neuronal activity of this nucleus is involved in the antidepressive effect, anti-cFos immunoreactivity might be useful for the evaluation of new antidepressants and abnormal neural activity in the nucleus might be involved in the etiology of depression itself. However, ROMK may not be the best target as a novel antidepressant, because TPN-RQ has an anxiogenic effect and impairs motor coordination. We expect that there should be a more suitable K^+^ channel among the 76 known subtypes, and further work is needed to identify the best target channel.

It remains unclear how TPN-RQ suppressed the increase in anti-c-Fos immunoreactivity in the lateral septum. It is unlikely that TPN-RQ directly suppressed the neural activity of the septum neuron. As shown in Fig 2D, ROMK channel conduct K^+^ current in a voltage-independent manner. If TPN-RQ directly blocked ROMK expressed in a neuron, it will be readily excitable through a positive shift of resting membrane potential and an inhibition of shunting effect and lead to the increase in the anti-c-Fos immunoreactivity. In situ hybridization analysis of the mRNA for the ROMK channel showed only modest expression in the lateral septum. In contrast, the highest expressions were observed in the hippocampus, cerebellum, and olfactory bulb, and somewhat lower expressions were observed in the cerebral cortex (especially piriform cortex), thalamus, striatum, pontine nucleus, dorsal and ventral parts of the medulla oblongata including locus ceruleus and inferior olive [46]. TPN-RQ significantly affected the anti-c-Fos immunoreactivity only in the lateral septum, without significantly affecting the immunoreactivity in these nuclei of higher expression. This possibility seems unlikely. It is also possible that the blockade of ROMK channel positively shifted the resting membrane potential and thereby induced the steady-state inactivation of the Na^+^ channel [47]. This again unlikely because the expression of mRNA for the ROMK channel was reported to be low in the lateral septum [46, 48]. Therefore, it is likely that TPN-RQ might activate another nucleus and thereby inhibit activity in the lateral septum indirectly. In fact, i.c.v. administration of TPN-RQ tended to increase c-Fos positive neurons in most nuclei we examined. Indeed, optogenetic stimulation of melanin-concentrating hormone neurons leads to a feedforward inhibition of the lateral septum neurons [49]. TPN-RQ might stimulate similar feedforward inhibition and thereby inhibit the nucleus. Alternatively, the lateral septum is known to be divided into 20 subregions [50], and a subset of neurons might be sensitively activated by the TPN-RQ, whereas others are insensitive. Moreover, Chee et al. [49] reported the GABAergic inhibitory neurons in the nucleus. There might be an inhibitory circuit to or even within the lateral septum neuron.

The lateral septum is known to be involved in anxiety- and depression-like behaviors [50]. Furthermore, the nucleus was reported to relay the information of stress to other nuclei, resulting in freezing behavior [40]. However, the suggested role of the lateral septum neurons in the anxiety-like behavior was controversial: some reports suggested that the nucleus suppresses anxiety behavior [50], whereas optongenetic stimulation of a subset of neurons, i.e., corticotoropin releasing factor rectptor-positive neurons, promoted anxiety-like behaviors [51]. TPN-RQ may inhibit the activity of the former anxiolytic neurons in the nucleus.

Depression is caused by mental stress. Previous studies have shown that acute stress increased the c-Fos immunoreactive neurons in the lateral septum [15, 33, 50]. Interestingly, it is reported that when mice were chronically subjected to mild stress, the number of c-Fos-positive cells and the plasma corticosterone levels were decreased nearly to the control level. These findings indicate that the c-Fos expression was suppressed in a stress-adapted condition in the nucleus. TPN-RQ administration might lead to this adaptation-like status, and thereby showed antidpressive effect. The administration of SSRI was reported to increase the neuronal activity of the lateral septum [50]. This again suggests that the difference in the mechanisms of action from the current antidepressant.

Generally, low molecular weight compounds are more suitable for medicines, because of their better drug-delivery characteristics than high molecular weight peptides. For this reason, we examined the blocking activities of short peptides and tried to identify the essential amino acids for blockade. Previous studies have shown the important role of basic amino acids (R7, K16, K17, K20, and K21) along with H12, Q13, and W15 of TPN for the blockade of ROMK [18, 41]. We synthesized various 8–15 amino acid residue peptides that contained most of these amino acids, e.g., TPN-RQ 7–13 + K (Table 1, Peptide # 22), TPN-RQ 5–17 (# 37), TPN-RQ 4–17 (#39), TPN-RQ 3-16(C5S) (# 41). Nevertheless, these peptides containing R7, R8, H12, Q13, and Lys at the C-terminus, none of them showed blocking activity, suggesting the requirement for a higher structure. As shown in Fig 1, the formation of two disulfide bonds determined the size of the ring and makes the ring inflexible. Our data showed that both the size and inflexibility are essential for the blockade. First, the reductions in activity in both the deletion or insertion of amino acids indicated the importance of the size of the ring. Second, the lack of activity of Cys-to-Ser mutants indicated the importance of inflexibility. For instance, TPN-RQ(C3S/C14S) (# 50), of which the size of the ring was supposed to be the same as the TPN-RQ, had no blocking activity.
